# Supplementary material for: HIVprotI: an integrated web based platform for prediction and design of HIV proteins inhibitors
Source: J Cheminform. 2018 Mar 9;10:12. doi: 10.1186/s13321-018-0266-y (PMC5845081; doi:10.1186/s13321-018-0266-y)
Supplement: Supplementary file 2 — Additional file 2. Source code of HIVProtI web server. [file 13321_2018_266_MOESM2_ESM.zip › HIVprotI_Source-code/hivprotI/weka-3-6-6/doc/weka/core/neighboursearch/balltrees/BallSplitter.html]

BallSplitter


---


|  |  |  |
| --- | --- | --- |
| **Overview** \| **Package** \| **Class** \| **Tree** \| **Deprecated** \| **Index** \| **Help** \| **Weka's home** | |  |
| **PREV CLASS**   **NEXT CLASS** | **FRAMES**    **NO FRAMES**     **All Classes** |  |
| SUMMARY: NESTED \| FIELD \| CONSTR \| METHOD | DETAIL: FIELD \| CONSTR \| METHOD |  |


---


## weka.core.neighboursearch.balltrees Class BallSplitter

```
java.lang.Object
  weka.core.neighboursearch.balltrees.BallSplitter
```

**All Implemented Interfaces:**: java.io.Serializable, OptionHandler, RevisionHandler

**Direct Known Subclasses:**: MedianDistanceFromArbitraryPoint, MedianOfWidestDimension, PointsClosestToFurthestChildren

---

``` public abstract class BallSplitter extends java.lang.Object implements java.io.Serializable, OptionHandler, RevisionHandler ```

Abstract class for splitting a ball tree's BallNode.

**Version:**
:   $Revision: 1.2 $

**Author:**
:   Ashraf M. Kibriya (amk14[at-the-rate]cs[dot]waikato[dot]ac[dot]nz)

**See Also:**: Serialized Form

---

**Constructor Summary**

`BallSplitter()`
  
          default constructor.

`BallSplitter(int[] instList,
Instances insts,
EuclideanDistance e)`
  
          Creates a new instance of BallSplitter.


| **Method Summary** | |
| --- | --- |
| `java.lang.String[]` | `getOptions()`             Gets the current settings of the object. |
| `java.lang.String` | `getRevision()`             Returns the revision string. |
| `java.util.Enumeration` | `listOptions()`             Returns an enumeration describing the available options. |
| `void` | `setEuclideanDistanceFunction(EuclideanDistance func)`             Sets the distance function used to (or to be used to) build the tree. |
| `void` | `setInstanceList(int[] instList)`             Sets the master index array containing indices of the training instances. |
| `void` | `setInstances(Instances inst)`             Sets the training instances on which the tree is (or is to be) built. |
| `void` | `setOptions(java.lang.String[] options)`             Parses a given list of options. |
| `abstract  void` | `splitNode(BallNode node, int numNodesCreated)`             Splits a node into two. |

**Methods inherited from class java.lang.Object**

`equals, getClass, hashCode, notify, notifyAll, toString, wait, wait, wait`

**Constructor Detail**

### BallSplitter

```
public BallSplitter()
```

:   default constructor.

---


### BallSplitter

```
public BallSplitter(int[] instList,
                    Instances insts,
                    EuclideanDistance e)
```

:   Creates a new instance of BallSplitter.

    **Parameters:**: `instList` - The master index array.: `insts` - The instances on which the tree is (or is to be) built.: `e` - The Euclidean distance function to use for splitting.


**Method Detail**

### listOptions

```
public java.util.Enumeration listOptions()
```

:   Returns an enumeration describing the available options.

    :   **Specified by:**: `listOptions` in interface `OptionHandler`
    :   **Returns:**: an enumeration of all the available options.

---


### setOptions

```
public void setOptions(java.lang.String[] options)
                throws java.lang.Exception
```

:   Parses a given list of options.

    :   **Specified by:**: `setOptions` in interface `OptionHandler`
    :   **Parameters:**: `options` - the list of options as an array of strings **Throws:**: `java.lang.Exception` - if an option is not supported

---


### getOptions

```
public java.lang.String[] getOptions()
```

:   Gets the current settings of the object.

    :   **Specified by:**: `getOptions` in interface `OptionHandler`
    :   **Returns:**: an array of strings suitable for passing to setOptions

---


### splitNode

```
public abstract void splitNode(BallNode node,
                               int numNodesCreated)
                        throws java.lang.Exception
```

:   Splits a node into two.

    :   **Parameters:**: `node` - The node to split.: `numNodesCreated` - The number of nodes that so far have been created for the tree, so that the newly created nodes are assigned correct/meaningful node numbers/ids. **Throws:**: `java.lang.Exception` - If there is some problem in splitting the given node.

---


### setInstances

```
public void setInstances(Instances inst)
```

:   Sets the training instances on which the tree is
    (or is to be) built.

    :   **Parameters:**: `inst` - The training instances.

---


### setInstanceList

```
public void setInstanceList(int[] instList)
```

:   Sets the master index array containing indices of the
    training instances. This array will be rearranged as
    the tree is built (or a node is split\_), so that each
    node is assigned a portion in this array which
    contain the instances insides the node's region.

    :   **Parameters:**: `instList` - The master index array.

---


### setEuclideanDistanceFunction

```
public void setEuclideanDistanceFunction(EuclideanDistance func)
```

:   Sets the distance function used to (or to be used
    to) build the tree.

    :   **Parameters:**: `func` - The distance function.

---


### getRevision

```
public java.lang.String getRevision()
```

:   Returns the revision string.

    :   **Specified by:**: `getRevision` in interface `RevisionHandler`
    :   **Returns:**: the revision


---


|  |  |  |
| --- | --- | --- |
| **Overview** \| **Package** \| **Class** \| **Tree** \| **Deprecated** \| **Index** \| **Help** \| **Weka's home** | |  |
| **PREV CLASS**   **NEXT CLASS** | **FRAMES**    **NO FRAMES**     **All Classes** |  |
| SUMMARY: NESTED \| FIELD \| CONSTR \| METHOD | DETAIL: FIELD \| CONSTR \| METHOD |  |


---
